# Post-radiation levator ani atrophy is associated with worse Low Anterior Resection Syndrome score after nonoperative management for locally advanced rectal cancer: a potential MRI biomarker

**DOI:** 10.3389/fonc.2025.1656898

**Published:** 2026-01-12

**Authors:** Rehema J. Thomas, Shrey Derasari, Sarah M. Palmquist, Nichole Samms, Lauren Andring, Emma B. Holliday

**Affiliations:** 1 Division of Radiation Oncology, The University of Texas MD Anderson Cancer Center, Houston, TX, United States; 2 McGovern Medical School at University of Texas Health Houston, Houston, TX, United States; 3 Division of Diagnostic Imaging, The University of Texas MD Anderson Cancer Center, Houston, TX, United States; 4 Department of Rehabilitation Services, The University of Texas MD Anderson Cancer Center, Houston, TX, United States; 5 St Charles Health System, Bend, OR, United States; 6 Department of Gastrointestinal Radiation Oncology, The University of Texas MD Anderson Cancer Center, Houston, TX, United States

**Keywords:** imaging biomarker, pelvic floor dysfunction, incontinence, radiation late effects, fibrosis

## Abstract

**Background:**

Nonoperative management is increasingly offered to patients who achieve a complete clinical response to neoadjuvant therapy for rectal cancer. However, long-term bowel dysfunction and fecal incontinence can occur, potentially due in part to post-radiation fibrosis and atrophy of the pelvic floor muscles. In this pilot study, we investigated post-radiation changes in the levator ani muscles and their association with long-term patient-reported bowel dysfunction and incontinence scores.

**Methods:**

Fifteen patients with rectal cancer treated with definitive chemoradiation followed by non-operative management were included. 2D and volumetric measurements of the levator ani were made on pre-treatment and 1-year post-radiation MRI. The changes in levator ani width and volume were correlated with the Low Anterior Resection Syndrome (LARS) and Fecal Incontinence Quality of Life (FiQOL) scores at least 1-year post treatment.

**Results:**

The 2D and volumetric measurements of the levator ani decreased between pre-treatment and 1-year post-radiation. (-21.0% and –21.7%, respectively; both P<0.01). The decrease in 2D levator ani measurements was positively associated with LARS score (Pearson r(13) = 0.55; P = .03) but not FiQOL score. The decrease in volumetric levator ani measurements was not significantly correlated with LARS or FiQOL scores.

**Conclusions:**

Decrease in 2D measurements of the levator ani from pre-treatment to 1-year post-radiation is correlated with higher LARS scores, indicating worse bowel function, perhaps due to radiation fibrosis and atrophy. This imaging biomarker may help to identify patients who could most benefit from pelvic floor physical therapy interventions. Further studies with larger cohorts are required to validate these findings.

## 1 Introduction

For decades, surgery with pre- or postoperative radiation and/or chemotherapy has been the mainstay of treatment for patients with locally advanced rectal cancer (LARC). However, the use of a nonoperative management strategy is increasing for patients who attain a complete clinical response (cCR) after radiation and/or chemotherapy (1). Interest in nonoperative management is driven by a desire to avoid surgery requiring a permanent ostomy or expected poor bowel function with sphincter-preserving surgery (2). Rates of Major Low Anterior Resection Syndrome (LARS), a constellation of symptoms that includes problems with frequency, urgency, and control of bowel movements, are nearly 50% when surgery requires a low anastomosis (3).

Although LARS was originally described as altered and problematic bowel function that occurs after rectal resection (4), studies suggest that approximately 25-33% of patients treated with definitive radiation and nonoperative management for rectal cancer can also develop Major LARS (5, 6). Bowel dysfunction, particularly fecal incontinence, after pelvic radiation is likely multifactorial (7). In the acute setting, radiation causes inflammation that leads to the development of fibrosis. This can result in issues with large bowel wall compliance, small bowel absorption, and persistent dysbiosis, causing diarrhea and urgency (8). Additionally, radiation-induced fibrosis, vascular changes, and nerve damage can lead to muscle atrophy within the pelvic floor muscles and sphincter complex (9).

Interventions such as pelvic floor physical therapy may improve post-treatment bowel dysfunction by isolating and strengthening pelvic floor muscles (10). However, tools for accurately assessing pelvic floor atrophy or damage remain limited. Magnetic resonance imaging (MRI) offers detailed visualization of soft tissues, including the pelvic floor, and may serve as a quantitative biomarker to detect radiation-induced atrophy (11). In this pilot study, we seek to evaluate 2D and volumetric levator ani measurements on pre- and 1-year post-treatment MRI among patients treated with radiation as a component of nonoperative management and correlate post-radiation MRI changes with LARS and patient-reported Fecal Incontinence Quality of Life (FIQoL) scores.

## 2 Methods

### 2.1 Patient population

This study was approved by the Institutional Review Board at The University of Texas MD Anderson Cancer Center (2020-0513). All patients provided informed consent to participate. We conducted a survey study of patients who completed pelvic radiation for rectal adenocarcinoma at our institution between 1/1/2017 and 12/31/2020 and were alive and without evidence of disease at the time of the survey administration in January 2022. Of 202 patients surveyed, 124 (61.4%) responded, and the results of their patient-reported quality of life outcomes have been reported elsewhere (12). For this pilot analysis of levator ani measurements, we selected the 15 patients from this cohort who had attained a cCR to chemoradiation and were treated with nonoperative management.

### 2.2 Treatment details

All patients were treated with long-course chemoradiation to a total dose of either 50.4Gy in 28 fractions or 50Gy in 25 fractions. Chemoradition was given alone or as a component of total neoadjuvant therapy depending on the stage and discretion of the multidisciplinary tumor board. Volumetric-modulated arc therapy (VMAT) or 3D conformal techniques were chosen at the discretion of the treating radiation oncologist. Concurrent capecitabine was given on the days of radiation. If patients were treated with induction chemotherapy followed by chemoradiation or by upfront chemoradiation, patients were assessed 6–8 weeks after chemoradiation by clinical exam, endoscopic examination, and pelvic MRI. Patients who were treated with upfront chemoradiation were observed if they had a cCR. Patients who had a near cCR were given consolidative chemotherapy and reassessed 2–6 weeks after the final cycle of chemotherapy. Once a cCR was confirmed endoscopically and radiogracphically, clinical and endoscopic exams were typically performed every 3 months, pelvic MRIs every 6 months, and CT abdomen and pelvis every 12 months for the first two years. Then, clinical exams, endoscopic exams, and pelvic MRIs were performed every 6 months until year 5, with CT abdomen and pelvis performed every 12 months until year 5.

### 2.3 Patient-reported bowel function

All patients in this study filled out a post-treatment patient-reported outcomes survey at least 18 months after completion of chemoradiation. The survey included two validated, patient-reported outcome measures of bowel function. First, the LARS score is calculated from five questions about incontinence, frequency, clustering, and urgency of bowel movements. LARS score ranges from 0-42, with higher scores indicating worse function. A score of 30–42 indicates the patient meets major LARS criteria (13). Second, the FIQoL score is calculated from 29 questions about lifestyle, coping/behavior, depression/self-perception, and embarrassment related to fecal incontinence. FIQoL score ranges from 4-20, with higher scores indicating better QoL (14).

### 2.4 Levator ani measurements

**Figure 1:**
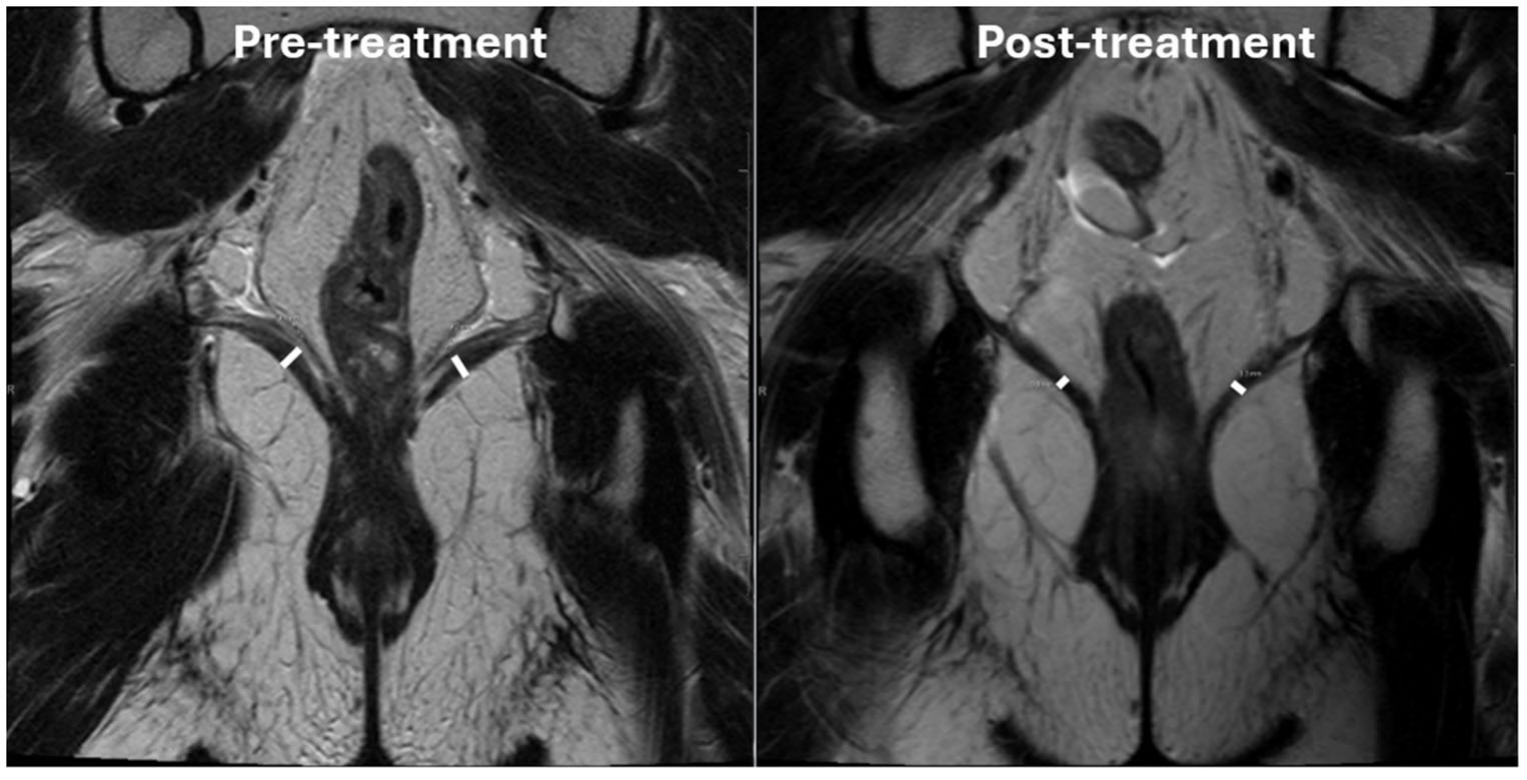
Pre-treatment and 12-month post-radiation MRI of a representative patient.

**Figure 2:**
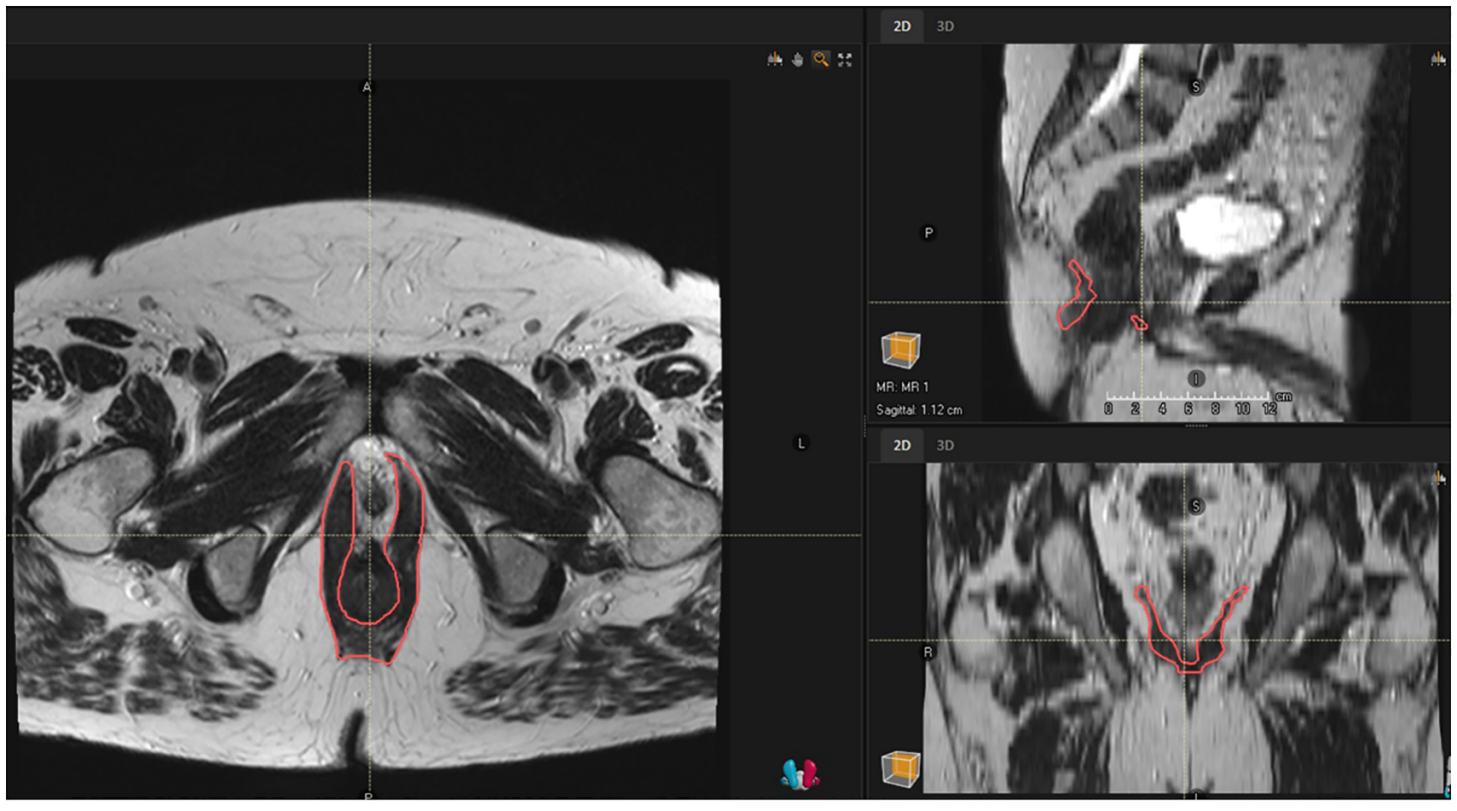
Axial, sagittal, and coronal view of the contoured levator ani muscles.

The levator ani muscles were assessed on pre-treatment and 12-month post-radiation MRI scans. MRIs were obtained on a 1.5 Tesla MRI scanner. T2-weighted coronal and axial sequences with 5mm slice thickness were used for measurements. 2D measurements (in mm) were made at the widest portion of the left and right levator ani muscles in the coronal plane by a specialized gastrointestinal and genitourinary diagnostic radiologist (SP) on both the pre-treatment and 12-month post-radiation scans (**Figure 1**). 2D measurements were standardized by following previously published measurement protocols for 2D levator ani assessment in the coronal plane (15). Volumetric measurements (in cc) of the levator ani were generated by importing the pre-treatment and 12-month post-radiation scans into RayStation version 12A (RaySearch Laboratories, 2022). The levator ani muscles were contoured in the axial plane by a radiation oncologist expert in lower gastrointestinal malignancies (EH) as a single structure that included the three paired muscle components: the iliococcygeus muscles originating from the ischial spine and attaching to the coccyx and anococcygeal raphe’, the pubococcygeus muscles originating from the back of the pubis extending along the anal canal and attaching to the coccyx and sacrum and the puboanalis muscles originating from the pubic bone, looping around the anorectal junction and inserting into the perineal body and rectum (**Figure 2**). 3D delineation of the levator ani muscles in the axial plane was performed by following previously published protocols and atlases as well (16, 17).

### 2.5 Statistical analysis

The Wilcoxon signed-rank test was used to assess potential differences in 2D and volumetric measurements pre-radiation versus post-radiation. The median and interquartile range (IQR) percent change were reported. The Pearson correlation coefficient was used to assess the strength and direction of the relationship between 2D and volumetric measurements, as well as between the levator ani measurements and both LARS and FIQoL scores. Significance was set at p<0.05.

## 3 Results

### 3.1 Demographic information

Fifteen patients were included in this pilot analysis. Nine men and six women. All patients were treated with long-course chemoradiation and were followed with close observation after attaining a complete clinical response. Demographic and clinical characteristics are outlined in **Table 1**.

**Table 1:** Demographic and clinical characteristics of patients treated with definitive radiation for locally advanced rectal adenocarcinoma.

Characteristics	Overall (N=15)
Age at radiation (median [IQR])	64 [53-71] years
Gender (N (%))	
Female	6 (40%)
Male	9 (60%)
Race (N (%))	
American Indian/Alaska Native	0 (0%)
Asian	1 (6.7%)
Black	0 (0%)
Pacific Islander	0 (0%)
White	14 (93.3%)
Ethnicity (N (%))	
Hispanic or Latino	2 (13.3%)
Non-Hispanic or Latino	13 (86.7%)
BMI (median [IQR])	28.4 [26.2-34.1]
Distance from the anal verge (median [IQR])	3 [1.75-6.45] cm
Clinical T-stage (N (%))	
T1	1 (6.7%)
T2	5 (33.3%)
T3	8 (53.3%)
T4	1 (6.7%)
Clinical N-stage (N (%))	
N0	7 (46.7%)
N1	7 (46.7%)
N2	1 (6.7%)
Mesorectal fascia involvement (N (%))	
>/=2mm	11 (73.3%)
<2mm	4 (26.7%)
Treatment type (N (%))	
Chemoradiation alone	8 (53.3%)
Total neoadjuvant therapy	
Chemotherapy → CRT	3 (20%)
CRT → Chemotherapy	4 (26.7%)
Radiation technique (N (%))	
3D Conformal	12 (80%)
Volumetric Modulated Arc Therapy	3 (20%)

### 3.2 Patient-reported bowel function

Post-treatment LARS and FiQOL scores represent long-term bowel function as the questionnaires were filled out a median [IQR] of 31.8 [19.2-47.9] months after completion of chemoradiation. No patient had an ostomy at the time of survey completion. No patient had experienced recurrent disease or had received any other cancer-directed therapy after chemoradiation. The median [IQR] LARS score was 24 [20-32]; seven (46.7%) patients met the criteria for Major LARS (score of 30-42). The median [IQR] FiQOL score was 15.3 [11.6-15.7]. Item-level survey responses for LARS and FiQoL are outlined in **Table 2** and **Supplementary Table**, respectively.

**Table 2:** Low Anterior Resection Syndrome questionnaire item responses for patients treated with definitive radiation for locally advanced rectal adenocarcinoma.

LARS questionnaire item	Response category, Patients, n (%)			
	More than 7 times per day (24 hours)	4–7 times per day (24 hours)	1–3 times per day (24 hours)	Less than once per day (24 hours)
How often do you open your bowels?	0 (0%)	4 (26.7%)	10 (66.7%)	1 (6.7%)
	Yes, at least once per week	Yes, less than once per week	No, never	
Do you ever have occasions when you cannot control your flatus (wind)?	6 (40%)	3 (20%)	6 (40%)	
Do you ever have any accidental leakage of liquid stool?	3 (20%)	4 (26.7%)	8 (53.3%)	
Do you ever have to open your bowels again within one hour of the last bowel opening?	5 (33.3%)	6 (40%)	4 (26.7%)	
Do you ever have such a strong urge to open your bowels that you have to rush to the toilet?	6 (40%)	9 (60%)	0 (0%)	

### 3.3 Levator ani measurements

The 2D and volumetric measurements of the levator ani decreased from pretreatment to 12 months post-treatment. The median [IQR] pretreatment and 12-month post-treatment 2D measurements of the right levator ani are 7.1 [5.1-8.0] mm and 4.8 [4.1-5.75] mm, respectively (P<.01). The median [IQR] pretreatment and 12-month post-treatment 2D measurements of the left levator ani are 5.2 [4.3-7.2] mm and 3.8 [3.25-6.1] mm, respectively (P<.01). The median [IQR] volume of the bilateral levator ani muscles pretreatment vs 12 months post-treatment are 44.3 [37.2- 54.1] cc vs 32.6 [29.1-42.7] cc, respectively (P<.01).

The median [IQR] percent difference between pretreatment and 12-month post-treatment 2D measurements (taken of the average between the right and left measurements) and volumetric measurements are -21.0% [-13.9--30.7%] and -21.7% [-9.5--28.0%], respectively. The 2D and volumetric measurements were moderately positively correlated (Pearson r(13) = 0.71; P<.01).

### 3.4 Association between levator ani measurements and patient-reported bowel function

The 2D (Pearson r(13) = 0.13; P = .63) and volumetric (Pearson r(13) = -0.18; P = .53) pretreatment levator ani measurements were not significantly correlated with the post-treatment LARS score. The decrease in 2D levator ani measurements between pretreatment and 12-month post-treatment MRI was moderately positively correlated with a larger (or worse) LARS score (Pearson r(13) = 0.55; P = .03) and not significantly correlated with the FiQOL score (Pearson r(13) = -0.37; P = 0.17). The decrease in volumetric levator ani measurements between pretreatment and 12-month post-treatment MRI was not significantly correlated with LARS score (Pearson r(13)= 0.25; P = .37) or FiQOL score (Pearson (r13) = -0.27; P = .33).

## 4 Discussion

In this pilot study, we show a decrease in 2D and volumetric measurements of the levator ani one year after definitive pelvic radiation for rectal adenocarcinoma. The demonstrated correlation between 2D levator ani measurements and LARS suggests MRI may be a useful biomarker for bowel dysfunction and incontinence. By integrating structural imaging with patient-reported outcomes, this work provides preliminary support for advancing personalized survivorship care strategies and will serve as the foundation for future studies.

We found significant reductions in both 2D and volumetric levator ani measurements at 12 months post-treatment, suggesting a lasting effect of radiation on pelvic floor atrophy. Radiation exposure is known to induce transforming growth factor-β (TGF-β) release and chronic inflammation, leading to sustained fibroblast activation and extracellular matrix deposition that replace normal tissue architecture with fibrotic tissue (18). Additionally, oxidative stress and DNA damage results in depletion of myogenic stem cells, impacting regenerative capacity and further propagating muscle atrophy (19). These pathophysiological mechanisms induced by radiation therapy result in structural changes in pelvic floor muscles (20, 21). While the optimal levator ani quantification strategy is unknown, the strong positive correlation between 2D and volumetric measurements in our study supports the potential utility of simplified linear assessments as a practical alternative to volumetric analysis. Notably, given that the changes were detected through routine baseline and surveillance imaging, clinical implementation may be feasible without additional imaging burden.

Among this cohort of 15 patients treated with definitive, long-course chemoradiation +/- chemotherapy for locally advanced rectal cancer, 7 (46.7%) had symptoms consistent with Major LARS at least 18 months from completion of therapy. This is somewhat higher than other reported series (5, 6). In a cohort of 221 patients treated with radiation and nonoperative management, the rate of major LARS was 25% (5). This variation may be attributed to the cumulative multimodal treatment burden associated with long-term chemoradiation in comparison to selective nonoperative strategies such as short-course radiotherapy. In a cohort of 33 patients similarly treated, the rate of major LARS was 33%. This study also included a dosimetric analysis, but there were no significant associations between dose to the anal sphincter complex and LARS score (6).

With respect to functional outcomes, a moderate correlation was observed between reductions in 2D measurements and worsening LARS scores, suggesting a trend towards clinically relevant functional decline in patients with a high degree of pelvic atrophy. In contrast, correlation with volumetric change and FiQOL scores were weaker and non-significant. These trends may reflect the multifactorial nature of post-treatment dysfunction. Differences between volumetric and 2D correlations may be attributed to greater sensitivity of single plane 2D measurements to focal levator ani thinning in regions most relevant with anorectal support. In contrast, volumetric measurements integrate multiple dimensions and may dilute localized atrophy measurements with functional implications. As evidenced by the questionnaires, patient-reported outcomes are influenced not only by structural factors but also by psychosocial and behavioral components. Notably, the FiQOL outcome assesses broader quality of life aspects, such as emotional and social well-being, which may not be directly linked to structural alterations compared to questions regarding stool leakage and urgency. These variations emphasize the need for multimodal assessments alongside imaging to evaluate both functional and psychosocial quality of life.

Therapeutic interventions, such as pelvic floor physical therapy, have demonstrated efficacy in managing radiation-induced dysfunctions such as incontinence, pain, and sexual dysfunction in pelvic cancer survivors (22, 23). Leveraging MRI findings to guide these interventions with early risk stratification could enhance their effectiveness, particularly for patients with significant muscle atrophy. Emerging tools with MRI through predictive models and radiomics may enhance the predictive value of imaging alongside functional response to guide and personalize treatment options (24, 25). While pretreatment levator ani width and volume were not significantly correlated with post-treatment LARS score, change post treatment may predict worse function. By identifying pelvic floor atrophy prior to symptom onset, clinicians may mitigate chronic pelvic dysfunction and the associated patient outcomes.

While this study provides promising insights, several limitations should be acknowledged. The small cohort size limits the statistical power and generalizability, and the lack of pre-treatment patient-reported functional assessments restricts the ability to assess functional changes over time. Furthermore, the absence of dosimetric data limits the evaluation of dose-response relationships with muscle volume loss. Finally, although we utilized published guidelines and atlases for the 2D and volumetric measurements in this pilot study, we only had one expert diagnostic radiologist recording 2D measurements and one expert radiation oncologist recording volumetric measurements. Measurements were recorded one time per patient and timepoint. Therefore, intra- and interobserver variability were not formally assessed. Future research should also explore advanced imaging techniques, such as diffusion tensor imaging, to assess microstructural changes in pelvic musculature. Establishing an MRI-based threshold for clinically significant atrophy would be helpful in defining criteria for risk stratification and early therapeutic intervention. Additionally, longitudinal studies are needed to understand the progression of atrophy and its relationship to functional outcomes and quality of life. We plan to use these pilot data to design future studies to risk-stratify patients for early therapeutic interventions for bowel dysfunction and assess response to treatment.

## Data Availability

The raw data supporting the conclusions of this article will be made available by the authors, without undue reservation.
