# Supplementary material for: Post-radiation levator ani atrophy is associated with worse Low Anterior Resection Syndrome score after nonoperative management for locally advanced rectal cancer: a potential MRI biomarker
Source: Front Oncol. 2026 Jan 12;15:1656898. doi: 10.3389/fonc.2025.1656898 (PMC12832774; doi:10.3389/fonc.2025.1656898)
Supplement: Supplementary file 1 [file Table1.docx]

**Supplemental Table**- Fecal Incontinence Quality of Life questionnaire item responses for patients treated with definitive radiation for locally advanced rectal adenocarcinoma

| **Due to accidental bowel leakage:** | **Most of the time** | **Some of the time** | **A little of the time** | **None of the time** |
| --- | --- | --- | --- | --- |
| I am afraid to go out | 0 (0%) | 1 (6.7%) | 4 (26.7%) | 10 (66.7%) |
| I avoid visiting friends | 0 (0%) | 1 (6.7%) | 1 (6.7%) | 13 (86.7%) |
| I avoid staying overnight away from home | 1 (6.7%) | 1 (6.7%) | 0 (0%) | 13 (86.7%) |
| It is difficult for me to get out and do things like going to a movie or church | 2 (13.3%) | 0 (0%) | 2 (13.3%) | 11 (73.3%) |
| I cut down on how much I eat before I go out | 1 (6.7%) | 2 (13.3%) | 4 (26.7%) | 8 (53.3%) |
| Whenever I am away from home, I try to stay near a restroom as much as possible | 2 (13.3%) | 3 (20%) | 2 (13.3%) | 8 (53.3%) |
| It is important to plan my schedule (daily activities) around my bowel pattern | 2 (13.3%) | 4 (26.7%) | 2 (13.3%) | 7 (46.7%) |
| I avoid traveling | 1 (6.7%) | 0 (0%) | 3 (20%) | 11 (73.3%) |
| I worry about not being able to get to the toilet in time | 1 (6.7%) | 4 (26.7%) | 7 (46.7%) | 3 (20%) |
| I feel I have no control over my bowels | 2 (13.3%) | 4 (26.7%) | 4 (26.7%) | 5 (33.3%) |
| I can’t hold my bowel movements long enough to get to the bathroom | 1 (6.7%) | 4 (26.7%) | 4 (26.7%) | 6 (40%) |
| I leak stool without knowing it | 0 (0%) | 2 (13.3%) | 3 (20%) | 10 (66.7%) |
| I try to prevent bowel accidents by staying very near a bathroom | 2 (13.3%) | 1 (6.7%) | 5 (33.3%) | 7 (46.7%) |
| **Due to accidental bowel leakage:** | **Strongly agree** | **Somewhat agree** | **Somewhat disagree** | **Strongly disagree** |
| I feel ashamed | 1 (6.7%) | 4 (26.7%) | 2 (13.3%) | 8 (53.3%) |
| I cannot do many of the things I want to do | 1 (6.7%) | 3 (20%) | 3 (20%) | 8 (53.3%) |
| I worry about bowel accidents | 1 (6.7%) | 5 (33.3%) | 3 (20%) | 6 (40%) |
| I feel depressed | 0 (0%) | 2 (13.3%) | 2 (13.3%) | 11 (73.3%) |
| I worry about others smelling stool on me | 0 (0%) | 2 (13.3%) | 2 (13.3%) | 11 (73.3%) |
| I feel like I am not a healthy person | 0 (0%) | 6 (40%) | 0 (0%) | 9 (60%) |
| I enjoy life less | 2 (13.3%) | 0 (0%) | 4 (26.7%) | 9 (60%) |
| I have sex less that I would like to | 2 (13.3%) | 4 (26.7%) | 5 (33.3%) | 4 (26.7%) |
| I feel different from other people | 2 (13.3%) | 3 (20%) | 3 (20%) | 7 (46.7%) |
| The possibility of bowel accidents is always on my mind | 0 (0%) | 4 (26.7%) | 5 (33.3%) | 6 (40%) |
| I am afraid to have sex | 0 (0%) | 2 (13.3%) | 5 (33.3%) | 8 (53.3%) |
| I avoid traveling by plane or train | 2 (13.3%) | 1 (6.7%) | 1 (6.7%) | 11 (73.3%) |
| I avoid going out to eat | 1 (6.7%) | 3 (20%) | 1 (6.7%) | 10 (66.7%) |
| Whenever I go someplace new, I specifically locate where the bathrooms are | 3 (20%) | 3 (20%) | 2 (13.3%) | 7 (46.7%) |
